# Celestial Anger

**Published:** 1874-12

**Authors:** 


Celestial Anger.—A Chinese laundryman, employed in a laundry situated in the alley between Chestnut and Pine and Sixth and Seventh streets, was sitting in the rear of the house yesterday trying to catch a breath of cool air. He was not successful in this, but he did catch a half of a watermelon rind, which some mischievous person across the valley dropped out of the window on top of his head. John got up in a very juicy and mad condition, and going into the house he returned with an old horse-pistol, which was about a foot and a half long, and which wouldn't ask much odds of a howitzer. The melon propellor was not in sight, but John knew he was somewhere in the building, and concluded to take his chances by shooting through the wall. He blazed away, and tore a hole the size of a brick in the wall of the building, just below the window. The report was like a clap of thunder, and it having died away, John went calmly into the house, with the remark, "Dam whitee boy; blowee dam head off!" No arrests were made.—*St. Louis Republican.*

